# Supplementary material for: Complexome profiling of the Chlamydomonas psb28 mutant reveals TEF5 as an early PSII assembly factor
Source: Plant Cell. 2025 Mar 17;37(6):koaf055. doi: 10.1093/plcell/koaf055 (PMC12164590; doi:10.1093/plcell/koaf055)
Supplement: koaf055_Supplementary_Data [file koaf055_supplementary_data.zip › psb28_interactive.zip!/plots/intensity/qualityPlots/peptides/FHL1_Cre03.g201100.t1.2.html]

### Cre03.g201100.t1.2

Trivial name: FHL1  
SeqCoverage [median]: 3.70  
Mass: 128.38 kDa  
  
 

| Sequence | MissedCleavage | Mass [Da] | wtIntensity | muIntensity | Score |
| --- | --- | --- | --- | --- | --- |
| AAEVALFGK | 0 | 904.50 | 267.64 | 0.00 | 67.03 |
| AVAFALTPGLEPIK | 0 | 1425.82 | 385.18 | 0.00 | 84.17 |
| LDDATLAAVSR | 0 | 1130.59 | 278.90 | 238.49 | 57.35 |
| LDVVDLIGR | 0 | 998.58 | 271.02 | 0.00 | 70.92 |
|  |  |  | 1202.7 | 238.5 |  |
